# The extinct Baltic amber genus *Propelma* Trjapitzin, a valid genus of Neanastatinae (Hymenoptera, Eupelmidae)

**DOI:** 10.3897/zookeys.283.4665

**Published:** 2013-04-03

**Authors:** Gary A. P. Gibson

**Affiliations:** 1 Agriculture and Agri-Food Canada, Canadian National Collection of Insects, Arachnids and Nematodes, K. W. Neatby Bldg., 960 Carling Avenue, Ottawa, Ontario, Canada, K1A 0C6

**Keywords:** Eocene, fossil, Dominican amber

## Abstract

The extinct Eocene Baltic amber genus *Propelma* Trjapitzin 1963 is removed from synonymy under *Eupelmus* Dalman 1820 (Hymenoptera, Eupelmidae, Eupelminae) and treated as a valid genus within Neanastatinae Kalina 1984 based on examination of the holotype female of *Propelma rohdendorfi* Trjapitzin. *Propelma rohdendorfi* is redescribed, illustrated by photomacrographs, and compared to other described extant and extinct genera of Neanastatinae. Taxonomic, morphological and geological diversity of Neanastatinae relative to Eupelminae and Calosotinae is also discussed relative to potential age of the subfamily.

## Introduction

Trjapitzin (1963) established *Propelma* based on *Propelma rohdendorfi* (Hymenoptera: Eupelmidae), which he described from a single female in Eocene Baltic amber. Gibson (1995) later synonymized *Propelma* under *Eupelmus* Dalman 1820 in a revision of the world genera of Eupelminae Walker 1833. The synonymy was based primarily on the lateral habitus drawing of *Propelma rohdendorfi* given by Trjapitzin (1963, fig. 1) without examining the holotype.

When Trjapitzin described *Propelma* he stated that it was similar to *Metapelma* Westwood 1835 in general habitus and size of the body, head shape, antennal structure, thorax, and presence of a long ovipositor. *Metapelma* is one of seven genera currently classified in Neanastatinae Kalina 1984, which along with Calosotinae Bouček 1958 and Eupelminae comprise the three recognized subfamilies of Eupelmidae. Of the seven neanastatine genera, four are extant, including *Eopelma* Gibson 1989, *Lambdobregma* Gibson 1989, *Metapelma* and *Neanastatus* Girault 1913. The other three genera, *Aspidopleura*, *Brevivula* and *Neanaperiallus*, were all described by Gibson (1999) from Baltic amber inclusions and are extinct. Description of the three extinct genera greatly expanded morphological limits of Neanastatinae and the new knowledge led me to re-examine Trjapitzin’s (1963) illustration of *Propelma rohdendorfi* and question the validity of synonymizing *Propelma* under *Eupelmus*. Resulting study of the amber holotype of *Propelma rohdendorfi* subsequently showed that Trjapitzin (1963) was correct in comparing *Propelma* with *Metapelma* relative to subfamilial affinities, and that *Propelma* represents a fourth, extinct, valid genus of Neanastatinae from Baltic amber. The purpose of this paper is to correct my erroneous synonymy and redescribe and illustrate *Propelma rohdendorfi* so that its classification is better established in Eupelmidae.

## Methods

**Figure 1.**
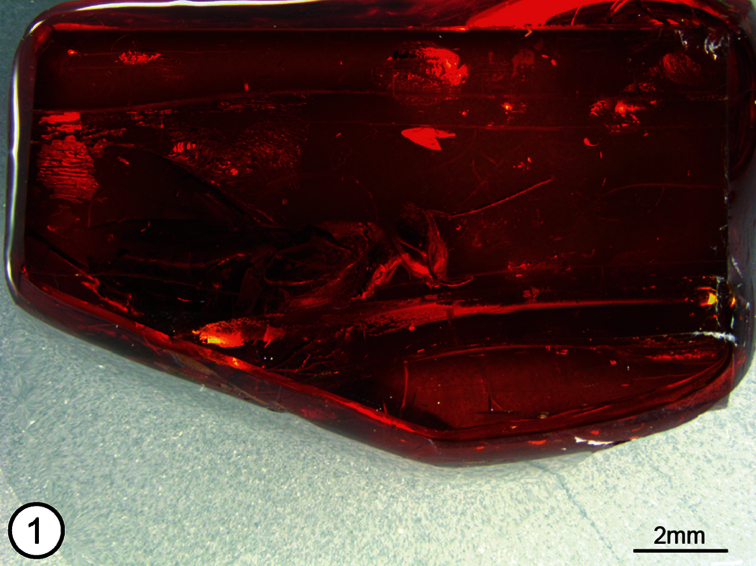
*Propelma rhodendorfi*, amber block bearing holotype female.

The description and photomacrographs are based on the holotype female of *Propelma rohdendorfi* [Holotype no. 364/360, Orlov Museum of Paleontology (formerly, Paleontological Institute of Russian Academy of Sciences), Moscow, Russia]. The holotype is in a mostly dark orange-colored block of amber (Fig. 1). It is complete except that an unknown length of the ovipositor sheaths are missing, as is most of the femur and tibia of the left hind leg and the apices of the femora and bases of the tibiae of the right middle and hind legs. The missing parts are because these projected beyond the sides of the polished amber block. The right side of the specimen, in particular, is clearly visible (Fig. 1), but artefacts prevent a direct ventral view of the mesosoma or the dorsal surface of the body beyond about the posterior angles of the axillae, and thickness of the amber prevents clear observation of the face. Images were taken with a Leica DFC 425C, 5 megapixel digital camera attached to a Leica Z16 APO macroscope. Serial images were combined using Zerene Stacker and digitally manipulated using Camera Raw and Adobe Photoshop 4 to enhance clarity. Images taken of the right side of the holotype for the plates of illustrations were flipped so that they face in the normal direction for specimen observation. All images except for Fig. 1, which illustrates color of the amber, are published in greyscale because this better facilitates differentiation of structures.

Terminology follows Gibson (1989, 1995) except terms used for the metanotum follow Heraty et al. (2013). Abbreviations used on the plates to indicate morphological features are: **acs** = acropleural sulcus; **amd** = anterolateral mesoscutal depression; **ams** = anterior, transverse region of mesoscutum; **car** = carina; **cer** = cercus; **cl_1–3_** = first, second, third clavomere; **fu_1, 8_** = first, eighth funicular; **gsp** = gastral spiracle; **map** = mesotibial apical pegs; **mbl** = membranous lobe; **mdr** = depressed triangular region of mesopectus; **mlp** = lateral panel of metanotum; **msp** = mesotibial spur; **Mt_8_** = eighth metasomal tergite; **mtp** = mesotarsal pegs; **mts** = mesotarsal setal line; **mtsa** = metanotal scutellar arm; **mtt** = metanotal trough; **pre** = lateral panel of prepectus; **psp** = propodeal spiracle; **ptl** = petiole; **sc_2_h** = mesoscutellar hook; **sc_3_** = metascutellum; **sp_2_** = mesothoracic spiracle; **syn** = syntergum (Mt_8_ + Mt_9_); **tgl** = tegula. Measurements of the antennomeres, fore wing venation, and metasomal tergites were all taken at the same magnification; measurements between square brackets are repeated from the original description.

## Results

### Neanastatinae

***Propelma* Trjapitzin, stat. rev.**

*Propelma* Trjapitzin, 1963: 89–91. Type species: *Propelma rhodendorfi* Trjapitzin, by original designation and monotypy. Synonymy under *Eupelmus* Dalman by Gibson (1995: 198).

#### Propelma rhodendorfi

Trjapitzin resurrected combination

http://species-id.net/wiki/Propelma_rhodendorfi

Figs 1 [Fig F2] –13

Propelma rhodendorfi Trjapitzin, 1963: 91–94. Holotype: female in Baltic amber. Label data: “Eupelmidae 364/360, *Propelma rohdendorphi*, Trjapitsyn 1963, Holotypus”.
Eupelmus rhodendorfi Trjapitzin; Gibson, 1995. New combination by inference through synonymy of *Propelma* under *Eupelmus*.

##### Redescription.

Female (Fig. 1). Length (anterior margin of head to posterior margin of syntergum in lateral view) = 7.9 mm [7.5]. Body mostly bright shiny orange (a reflection artefact, original color apparently mostly or entirely dark based on some regions of the body such as part of tegula (Fig. 6: tgl) and gastral tergites (Figs 12, 13)).

**Figures 2–7. F2:**
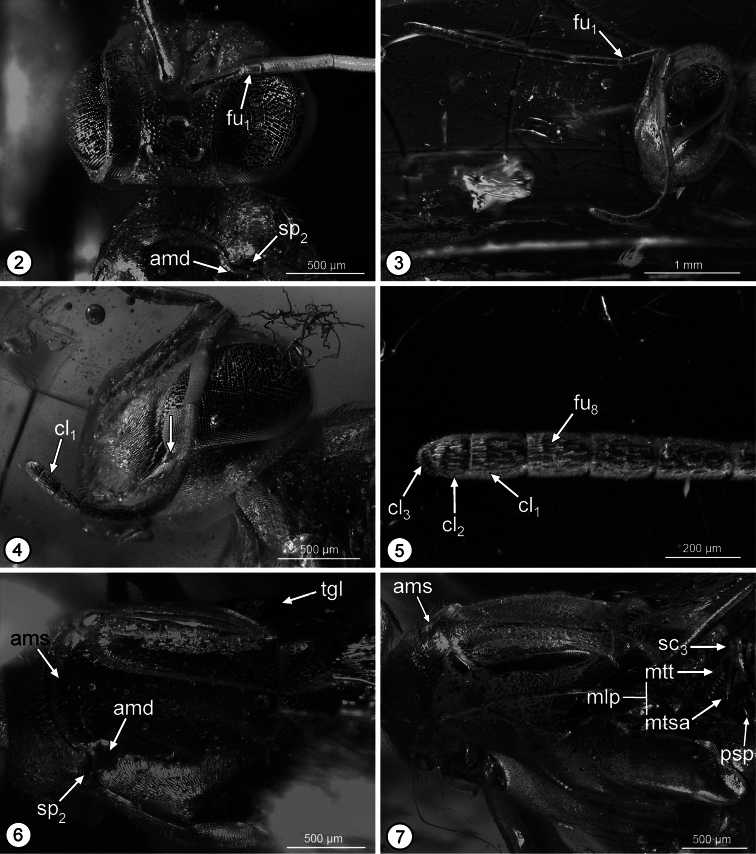
*Propelma rhodendorfi*: **2** head, frontodorsal view **3** head and antennae, lateral view **4** head and right antenna, lateral view **5** apical three funiculars and clava **6** mesoscutum, dorsal view **7** mesosoma, dorsolateral view. See Methods for abbreviations for structural features (arrow on Fig. 4 points to triangular region differentiated by putatively bifurcate malar sulcus).

Head in frontal view almost as wide as high, with ventral margin of torulus in line with lower orbits and with convex, dorsally tapered interantennal region separating distinct scrobes over at least ventral half of scrobal depression (Fig. 2); scrobal depression inverted U-shaped with minimum distance between lateral margin and inner orbit about 0.4× maximum diameter of anterior ocellus, abruptly margined dorsolaterally to within about one maximum diameter of anterior ocellus where slight change in curvature differentiates more obscure dorsal margin from bare, similarly finely coriaceous, slightly concave region below anterior ocellus (Fig. 2) such that under some angles of view scrobal depression superficially appears to extend to ocellus; upper parascrobal region and frontovertex minutely coriaceous-granular with minute setiferous punctures; lower parascrobal region and gena more vertically coriaceous-alutaceous with short white setae similar to upper parascrobal region, frontovertex and interantennal region. Head in lateral view (Fig. 4) with vertex smoothly rounded into occiput; almost twice as high as maximum length at level of toruli; malar sulcus appearing bifurcate near lower orbit, delineating small triangular region below posteroventral orbit (Fig. 4: arrow) (see discussion); eye about 1.6× as high as wide, superficially bare, but with very short, sparse setae. Head in dorsal view with minimum distance between inner orbits about 0.3× width of head; anterior ocellus slightly transverse, with maximum diameter equal to distance between its outer margin and inner orbit, and slightly greater than maximum diameter of posterior ocellus (Fig. 2); POL: LOL: OOL: maximum diameter of anterior ocellus = 1.0: 0.8: 0.3: 1.0. Antenna (Figs 3, 4) with scape slightly widened distally, ventral margin straight; length of pedicel plus flagellum about 1.7× width of head; flagellum and clava slender, of similar width throughout (Fig. 3); length of scape = 4.0 (approximate); length of pedicel = 2.0; funicle 8-segmented, with fu_1_ longer than wide, but much shorter than pedicel or fu_2_ (Figs 2, 3), relative lengths of funiculars = 0.7: 2.7: 2.2: 1.6: 1.1: 1.0: 0.9: 0.9; clava 3-segmented (Fig. 5), length = 1.5, basal clavomere slightly longer than cl_2_+cl_3_, separated from cl_2_ by distinct transverse suture (Figs 4, 5), but cl_3_ as tiny apical micropilose sensory region delineated by extremely fine, sinuate suture such that under some angles clava superficially 2-segmented (Fig. 4) (relative length of clavomeres = 8:5:2); [length:width ratios of pedicel to clava = 30:9, 10:7, 40:8, 33:8, 23:8, 16:7, 15:8, 12:9, 12:9, 22:9]; flagellum with numerous multiporous plate sensilla in multiple rows per flagellomere and with very short, inconspicuous setae (Figs 4, 5).

**Figures 8–13.**
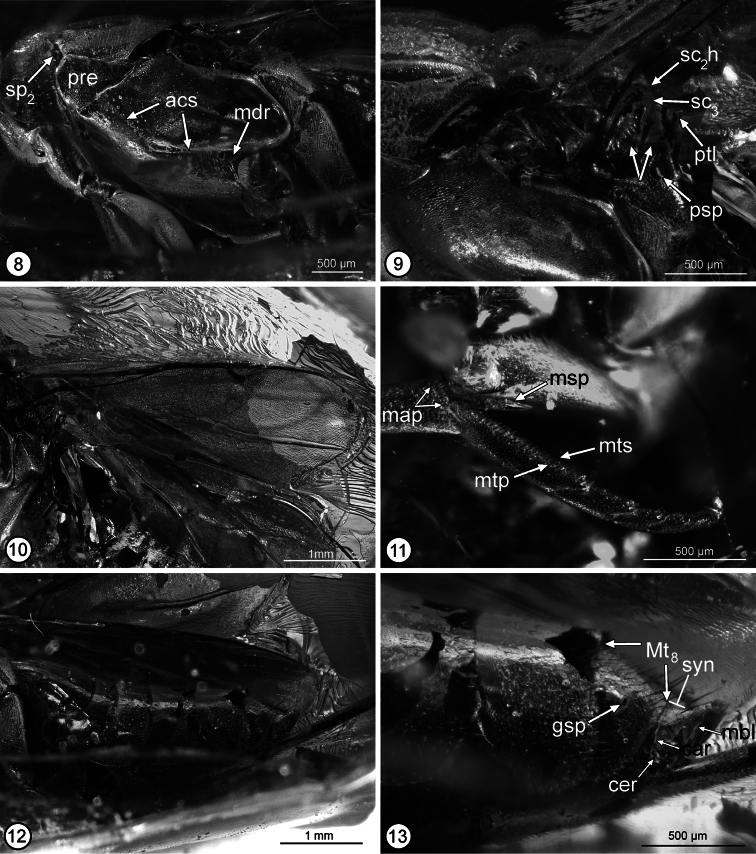
*Propelma rhodendorfi*: **8** mesosoma, lateral view **9** mesoscutellar-axillar complex to base of metasoma, lateral view **10** fore wing **11** apex of mesotibia and mesotarsus **12** gaster, lateral view **13** Mt6 to apex of metasoma, lateral view. See Methods for abbreviations for structural features (arrows on Fig. 9 point to sulci on propodeal callus and metanotal scutellar arm).

Pronotum uniformly sclerotized, dorsally convex in transverse plane and flat mediolongitudinally, hence without differentiated collar and neck (Fig. 7); more or less bell-shaped, sinuately narrowed anteriorly, with incurved posterior margin (Figs 2, 6) (holotype with pronotum rotated anteroventrally such that dorsal surface at obtuse angle relative to mesonotum (Fig. 7) and exposing convex, asetose, transverse anterior part of mesoscutum (Figs 6, 7: ams) between short lateral depression (Figs 2, 6: amd) posterior to each mesothoracic spiracle (Figs 2, 6: sp_2_), which accept dorsolateral angles of pronotum when this rotated horizontally in same plane as mesoscutum); finely coriaceous-granular to coriaceous-alutaceous with short black setae except for a line of longer setae along posterior margin. Mesoscutum slightly wider than long, with ridge-like medial elevation extending between exposed transverse anterior portion and transscutal articulation (Figs 6, 7), and with lateral lobes evenly convex; finely coriaceous, with short dark setae similar to pronotum. Mesoscutellar-axillar complex (Figs 6, 7, 9) with axillae transverse-triangular with contiguous inner angles, convex with abruptly angled, oblique, strongly crenulate posterior surfaces forming scutoscutellar sutures; mesoscutellum similarly convex as axillae, teardrop-shaped (cf. Gibson 2009, fig. 13), posteriorly tapered with apex curved down as short hook-like medial protrusion (Fig. 9: sc_2_h) over metascutellum (Fig. 9: sc_3_), apparently uniformly setose; axillula with dorsal margin carinate. Tegula (Fig. 6: tgl) triangular with almost truncate posterior margin. Prepectus (Fig. 8: pre) with lateral panel flat, anteriorly not protruding anterior of level of mesothoracic spiracle (Fig. 8: sp_2_), triangular, 1.4× as long as high basally, with dorsal and ventral margins convergent to narrowly rounded posterior angle; finely coriaceous, bare. Acropleuron (Figs 7–9) extended posteriorly to metapleuron and anteroventral margin of metacoxa between meso- and metacoxa, without exposed mesepimeron; acropleural sulcus (Fig. 8: acs) horizontal ventrally to level about equal with apex of tegula, where curved dorsally as shallower, oblique groove to level of about middle of prepectus, bare, minutely meshlike coriaceous-reticulate within about anterior quarter but more minutely meshlike coriaceous mesally, finely meshlike coriaceous posteriorly, and more elongate striate-coriaceous posterodorsally (Figs 8, 9). Mesopectus (Fig. 8) uniformly setose below acropleural sulcus; posteriorly with small, depressed, triangular region (Fig. 8: mdr) between its posterodorsal margin, acropleural sulcus, and anterolateral margin of mesocoxa; ventrally with posterior margin transverse, abutting anterior margins of mesocoxae, with sulcate discrimen but without transepisternal sulcus. Metanotum (Figs 7, 9) composed of median, slightly raised, flat, strongly transverse metascutellum (Figs 7, 9: sc_3_) and metanotal lateral panels (Fig. 7: mlp), each lateral panel broadened laterally and differentiated by transverse crenulate groove into anterior metanotal trough (Fig. 7: mtt) and posterior metanotal scutellar arm (Fig. 7: mtsa); metascutellar arm with about inner half developed as carinate ridge along posterior margin of lateral panel, and with what appears as a fine, obliquely longitudinal sulcus (Fig. 9: left arrow) near middle of outer broadened part; posterior margin of metanotum between lateral panels raised slightly above anterior margin of propodeum. Metapleuron (Fig 8, 9) elongate-triangular with posterior margin straight and anterior margin slightly sinuate, uniformly setose with white setae similar to callus. Fore wing (Fig. 10) hyaline, uniformly setose with dark setae, without speculum or linea calva; cc: mv: stv: pmv = 9.4: 3.9: 1.1: 6.2 [smv/mv/stv/pmv =13/5.5/one-third mv/8.0]; stigmal vein apically curved, tapered into short uncus without differentiated stigma. Middle leg in holotype with mesocoxa rotated slightly anteriorly (Fig. 8), its posterior margin separated from anteroventral margin of metacoxa, with outer surface finely, obliquely striate, bare; mesotibia with row of at least eight short, black pegs anteroapically (Fig. 11: map) and with robust mesotibial spur (Fig. 11: msp) about 1.7× as long as apical width of tibia; mesotarsus with line of setae (Fig. 11: mts) along posterior margin and line of short black pegs (Fig. 11: mtp) along anterior margin of basal four tarsomeres, and basitarsus in lateral view with maximum length slightly greater than combined length of remaining four tarsomeres (about 5:4). Hind leg with exterior surface of coxa completely, densely setose similar to metapleuron; tibia and tarsus not compressed. Propodeum (Fig. 9) with posterior margin broadly, shallowly incurved, with what appears as a fine longitudinal sulcus (Fig. 9: right arrow) mesad propodeal spiracle (Fig. 9: psp) in line with sulcus on metanotal scutellar arm, the putative sulcus (see discussion) thus differentiating medial plical region from callus; plical region strongly transverse, flat, asetose, and apparently very finely coriaceous; callus (Figs 8, 9) anteriorly setose to level of inner margin of propodeal spiracle and posteriorly setose to outer margin of spiracle, with white setae; spiracle (Figs 7, 9: psp) large, transverse-oval, with distance between anterior margin of spiracular rim and anterior margin of propodeum almost 3× distance between posterior margin of rim and propodeum.

Metasoma (Fig. 12) about 0.9× combined length of mesosoma and head; petiole (Fig. 9: ptl) a strongly transverse dorsal strip; in lateral view medial length of tergites from petiole to syntergum = 0.3: 5.2: 1.5: 2.0: 2.8: 3.1: 2.3: 0.8 [Mt_2_–syntergum = 43: 11: 16: 23: 29: 18: 3+4]; Mt_2_ to basal half of Mt_6_ comparatively sparsely setose with dark setae, but Mt_7_ and apical half of Mt_6_ (Fig. 13) more densely setose with longer dark setae, the setae longest in apical half of Mt_7_, and tergites dorsally very finely meshlike coriaceous; Mt_7_ with gastral spiracle (Fig. 13: gsp) cone-like protuberant anterolaterally; Mt_8_+Mt_9_ fused into syntergum (Fig. 13), but with transverse carina (Fig. 13: car) extending at least partly between cerci, the carina continuous along anterior and outer margins of cercus (Fig. 13: cer), with cercus at about mid-length of syntergum, and syntergum sparsely setose but with a few longer, more conspicuous dark setae along posteromesal margin; triangular membranous lobe (Fig. 13: mbl) extending from posterior margin of syntergum, but not extended into anal filament. Ovipositor sheaths tubular, conspicuously exerted, but of unknown length.

## Discussion

*Propelma* is assigned to Neanastatinae based on pronotal structure, mesopectus posteroventrally abutting the mesocoxal bases without a membranous region anterior to each coxa, and mesotarsal peg pattern, all of which are diagnostic of the subfamily (Gibson 1989, 2009). The scutoscutellar suture is also crenulate similar to extant and extinct species of *Metapelma* (Gibson 2009, fig. 13) and some species of *Lambdobregma* (Gibson 2009, fig. 15) (extant) as well as *Aspidopleura* (Gibson 2009, fig. 49) and *Brevivula* (Gibson 2009, fig. 39) (extinct), and the scutellum apically is curved ventrally into a small hook-like process over the metanotum similar to extant species of *Lambdobregma* (Gibson 2009, figs 15, 16) and *Neanastatus* (Gibson 2009, fig. 21) as well as *Brevivula* (Gibson 2009, fig. 40).

*Propelma* keys to couplet 6 (*Brevivula* and *Lambdobregma*) using the key to genera of Neanastatinae in Gibson (2009). It differs from both genera in having the fore wing uniformly setose (Fig. 10) rather than with a linea calva (cf. Gibson 2009, figs 6, 42) and by having the prepectus as an isosceles triangle, only about 1.3× as long as high with the dorsal and ventral margins convergent to a narrowly rounded apex (Fig. 8: pre), rather than being conspicuously elongate-triangular and tapered into an acute angle (cf. Gibson 2009, figs 21, 41). It also differs from *Lambdobregma* by the absence of a transepisternal sulcus (Fig. 8; cf. Gibson 1989, fig. 92), and from *Brevivula* by having the mesoscutal lateral lobes evenly convex (Figs 6, 7) rather than carinately margined (cf. Gibson 2009, fig. 39).

Trjapitzin (1963) interpreted Mt_8_ and Mt_9_ as separate tergites in *Propelma rohdendorfi*, but these are certainly fused into a syntergum (Fig. 13: syn). He also interpreted the bare triangular region at the apex of the syntergum as Mt_10_. However, comparison with extant taxa suggests that this region is a membranous lobe (Fig. 13: mbl) homologous with the anal filament of some extant chalcidoids with comparatively long ovipositor sheaths (cf. Gibson 1989, figs 148, 153). Trjapitzin (1963) also noted that the malar sulcus appears bifurcate in the holotype (Fig. 4), though this and what appears like a continuous sulcus across the propodeum and metanotum (Fig. 9: arrows) may more likely represent artefacts of preservation rather than real structures.

The recognition of *Propelma* as a valid genus of Neanastatinae results in four extinct genera described from Baltic amber (*Aspidopleura*, *Brevivula*, *Neanaperiallus* and *Propelma*) and four extant genera, of which one (*Lambdobregma*) is restricted to the New World, one (*Eopelma*) is restricted to the Oriental region, one (*Neanastatus*) is Old World in distribution, and one (*Metapelma*) is more widely distributed throughout both the Old and New World. *Metapelma* is also the only extant genus with a described extinct species, *Metapelma archetypon* Gibson (2009), from Baltic amber. Pike (1995) and Perrichot et al. (2010) both questionably recorded a species of Eupelmidae from Grassy Lake Canadian Cretaceous and Charentese French Cretaceous amber, respectively. I have been unable to obtain these inclusions for examination, but the earliest known verifiable eupelmids are all Neanastatinae from Baltic amber. Extant Neanastatinae is far less diverse, measured either by number of described species (82) or genera (4), than either Calosotinae (152/8) or Eupelminae (729/33) (Noyes 2012). It is unknown whether the absence of Eupelminae and Calosotinae from Baltic amber is because these two clades diversified more recently than the Eocene age (55–34 mya) of Baltic amber (Weitschat and Wichard 2010) or because some biological factor such as host taxon, host stage or host habitat favoured fossilization of Neanastatinae over the other two groups in Baltic amber resin. Eupelminae are recorded from Dominican amber (20–30 mya), including one taxon identified as the extant genus *Zaischnopsis* Ashmead (Wu 1997, Poinar and Poinar 1999), but the diversity of eupelmids in Dominican amber has yet to be described or analyzed. Eupelminae have extremely diverse host biologies (Gibson 1997), though relatively few, including *Zaischnopsis* (Gibson 1995), are parasitoids of wood-boring beetles. Most Calosotinae are parasitoids of wood-boring Coleoptera, which might be thought to favour fossilization of individuals in amber unless the amber producing trees in Baltic forests lacked suitable hosts. Members of *Metapelma* are also parasitoids of wood-boring Coleoptera and, as noted above, one species has been described from Baltic amber. Members of *Neanastatus* are primary or secondary parasitoids of Cecidomyiidae (Diptera), and at least some members of *Lambdobregma* could be egg parasitoids based on a single putative rearing of *Lambdobregma schwarzii* (Ashmead) from cricket eggs (Orthoptera: Grylloidea) (Gibson 1989). Such a diverse host range among so few extant genera and the greater morphological diversity encompassed by the extinct and extant genera (Gibson 2009) compared to Calosotinae and Eupelminae could indicate Neanastatinae is a comparatively old lineage. Munro et al. (2011, Fig. 1) retrieved Neanastatinae as a relatively basal clade of Chalcidoidea with no close relationships to Calosotinae or Eupelminae using strictly molecular evidence. Using combined molecular and morphological evidence, Heraty et al. (2013) either retrieved Neanastatinae + Calosotinae as the sister-group of Eupelminae (likelihood analysis, fig. 10) or as a paraphyletic assemblage relative to Cynipencyrtidae and Tanaostigmatidae (parsimony analysis, fig. 9).

## Supplementary Material

XML Treatment for Propelma rhodendorfi
